# The impact of COVID-19 pandemic on training and mental health of residents: a cross-sectional study

**DOI:** 10.1186/s12909-021-02655-2

**Published:** 2021-04-13

**Authors:** Eman Alshdaifat, Amer Sindiani, Wasim Khasawneh, Omar Abu-Azzam, Aref Qarqash, Hassan Abushukair, Nail Obeidat

**Affiliations:** 1 Department of Obstetrics and Gynecology, Faculty of medicine, Yarmouk University, P.O.Box: (566), Irbid, 21163 Jordan; 2 Department of Obstetrics and Gynecology, Faculty of medicine, Jordan University of Science and Technology, Irbid, Jordan; 3 Department of Pediatrics and Neonatology Faculty of medicine, Jordan University of Science and Technology, Irbid, Jordan; 4 Department of Obstetrics and Gynecology, Faculty of medicine, Mutah University, Al-karak, Jordan; 5 Faculty of medicine, Jordan University of Science and Technology, Irbid, Jordan

**Keywords:** COVID-19, Mental health, Residency, Training program

## Abstract

**Background:**

Residency programs have been impacted by the Coronavirus disease 2019 (COVID-19) pandemic. In this study we aim to investigate and evaluate the impact of the pandemic on residents as well as residency training programs.

**Methods:**

This was a cross-sectional study including a survey of 43 questions prepared on Google forms and electronically distributed among a convenience sample of residents training at a tertiary center in North Jordan during the COVID-19 pandemic. Data were collected in the period between October 30th and November 8th of 2020. The survey included questions that addressed the impact of the pandemic on residents’ health as well as training programs. The study participants included residents in training at KAUH in 2020 and were stratified according to the type of residency program (surgical residents (SRs) and non-surgical residents (NSRs)). Statistical methods included descriptive analysis, Chi-square or Fisher’s exact test, Mann Whitney U test, and Cramer’s V and r statistics as measures of effect sizes.

**Results:**

Of all 430 residents, 255 (59%) responded to the survey. A total of 17 (7%) of residents reported being infected with COVID-19 and a significant difference was reported between SRs and NSRs (10% vs 4%, V = .124 “small effect” (95% CI; .017–.229), *p* = 0.048). Approximately, 106 (42%) reported a decrease in the number of staff working at the clinic and 164 (64%) reported limited access to personal protective equipment during the pandemic. On a 4-point Likert scale for the feeling of anxiety, the median was 2 (2–3 IQR) in the NSRs group, vs 2 (1–2 IQR) in the SRs groups, with the NSRs being more likely to feel anxious (r = 0.13 “small effect” (95% CI; 0.007–0.249), *p* = .044). Similarly, the proportion of residents who reported feeling anxious about an inadequacy of protective equipment in the work area was significantly greater in the NSRs group (90.3% vs 75.2%; V = .201 “small effect” (95% CI; .078–.313), *p* = .001), as well as the proportion of residents who reported feeling increased stress and anxiety between colleagues being also significantly higher in the NSRs group (88.1% vs 76%; V = .158 “small effect” (95% CI; .032–.279), *p* = .012).

**Conclusion:**

The burden of the ongoing pandemic on the mental health status of residents is very alarming and so providing residents with psychological counseling and support is needed. Also, critical implications on the flow of residency training programs have been noticed. This necessitates adapting and adopting smart educational techniques to compensate for such limitations.

**Supplementary Information:**

The online version contains supplementary material available at 10.1186/s12909-021-02655-2.

## Background

Since the declaration of COVID-19 outbreak as a pandemic by the World Health Organization (WHO) in March 2020, the disease caused by The severe acute respiratory syndrome coronavirus 2 (SARS-CoV-2) has quickly spread all over the world [1–3]. In Jordan, the Ministry of Health confirmed the first COVID-19 case on March 2, 2020 [4]. Upon confirming the first case of COVID-19 in Jordan, the government enforced national measures to limit the spread of the infection starting with activating defense laws which were followed by imposing a national curfew on March 21, 2020, and on March 15, 2020 mandatory quarantine for air travelers was imposed before completely shutting down the airport on March 17, 2020. The number of confirmed cases in Jordan since the beginning of the pandemic is 234,353 confirmed cases and 2960 deaths as of December 5, 2020 [5, 6].

Following the spread of the disease, most academic institutions all over the world have suspended on-campus teaching programs and shifted toward distant learning programs. In this regard, clinical training programs for medical students and residents have been dramatically affected as they require hands-on interaction with patients which originally takes place in healthcare facilities; mainly hospitals. Also, a concerning knowledge deficit as well as fear of COVID-19 has been reported in a survey study among medical students and healthcare workers in previous studies, this also highlights the need for adequate training and focused education if their involvement in combating this pandemic is to be taken seriously [7, 8]. In a recent study carried out in Saudi Arabia which is a nearby country with quite similar setting as in Jordan, an 84.6% reduction in training activities was reported in residency training programs [9]. Other programs deploy residents and fellows as primary care doctors in screening facilities, critical care units or in emergency rooms [10]. In our hospital, King Abdullah University Hospital (KAUH) which is the academic center affiliated with Jordan University of Science and Technology (JUST), the impact of the pandemic on residency training programs was further complicated due to the fact that KAUH was designated by the Jordanian Ministry of Health as the main center for treating COVID-19 patients and one of seven centers performing PCR testing for suspected cases [11].

This in return, restricted the medical care provided by the hospital which was limited to only emergency admissions and major elective surgeries in the midst of COVID-19 cases surge in Jordan. Within these settings and as a result to the decrease in surgical and clinical practice, residents’ training has been seriously affected. Evaluating and understanding this impact will definitely aid in better assessment of the gains and losses of such modified residency programs adopted in light of the ongoing pandemic. In this questionnaire-based cross-sectional study we aimed to study and evaluate the impact of the COVID-19 pandemic on the residents and the training programs at KAUH. Primary outcomes were to assess the effect of the pandemic on the daily workflow and clinical experience as well as on the mental health of residents. Secondary outcomes were the prevalence of the COVID-19 infection among the residents and their family members, the impact of the pandemic on the residents’ social life and mental health and their coping with the overall pandemic.

## Methods

### Study design and sampling

This is a single institution cross-sectional study, in which a self-administered online-based questionnaire was sent to all residents (*n* = 430) at King Abdullah University Hospital (KAUH). KAUH is a 620-bed academic tertiary hospital affiliated with Jordan University of Science and Technology that hosts nineteen residency programs recruiting nearly 100 residents each year. A convenience sampling technique was utilized in this study, as the form was distributed online to all KAUH residents. The questionnaire was set on Google forms. The inclusion criteria included any resident who was in training at KAUH from the time of the COVID-19 pandemic onset in Jordan(March 2020) till the time of conducting the study in November 2020.

The participants were then stratified into two groups, surgical residents group (SRs) which included ENT surgery, general surgery, neurosurgery, obstetrics & gynecology, ophthalmology, orthopedic, and urology residents, and a non-surgical residents group (NSRs) which included all other specialties.

### Instrument development and data collection

The survey included 43 questions in English which is the official language of education at KAUH, investigating the impact of COVID-19 pandemic on residency training programs at KAUH in Irbid, Jordan. To ensure the questionnaire validity, the questions were distributed to a group of experts to give their judgment about the clarity and comprehension of questions and evaluation of the survey structure. The questionnaire was pilot-tested on 15 residents before a final version was developed and approved. Both closed and open-ended questions were included, of which seven questions concerning resident’s mental health had a four-point Likert format, and four questions that allowed more than one answer. Questions addressed demographic data, training details before and during the pandemic, impact of the pandemic on residents whether related to their residency programs or to the health-related burden of the pandemic. This burden was assessed in terms of both direct impacts as in disease contraction (in residents and their close relatives or contacts) or indirect impact as in resulting mental health effects of the pandemic (depression symptoms, stress, anxiety). This questionnaire aimed to confirm some the previously reported negative burden of the pandemic on clinical and academic outcomes at training programs also the overall effect of the type of residency program was explored in our sample [12]. The questionnaire was initially distributed to all included residents on October 30th. Follow up reminders were sent after four and 7 days. Data collection was completed on November 8th 2020.

### Statistical analysis

Data were analysed using IBM SPSS version 26 [13]. Descriptive measures for categorical data included counts and proportions (%). Likert-scale data were considered as ordinal and were assigned scores, starting with “Never = 0” and ending with “Always = 3”, and thus were described as medians and IQRs.

Chi-square tests, or Fisher’s exact tests if one cell count was less than 5, were used to analyze associations between nominal variables. While Likert-scale data were analyzed using Mann Whitney U tests for differences in distributions. Cramer’s V and r statistics with their 95%CI were calculated as effect size measures for nominal and Likert-scale data respectively, considering (0.10 - < 0.30) as small effect, (0.30 - < 0.50) as a medium effect, and (≥0.50) as a large effect for both, as described by Cohen and Kim [14, 15]. 95% CIs of Cramer’s V statistics were calculated using the bootstrapping method, setting the number of replicate samples to 1000.

A two-sided *p*-value of ≤0.05 was considered statistically significant. The internal consistency of Likert-type questions related to mental health was measured using Cronbach’s alpha, and a minimum value of 0.7 was set as an acceptable level of reliability. This study was done in accordance with the STrengthening the Reporting of OBservational studies in Epidemiology (STROBE) guidelines [16].

### Ethical consideration

This study was approved by Institutional Review Board (IRB) at Jordan University of Science and Technology (105/136/2020). In addition, informed consent was obtained from all participants.

## Results

### General demographics

Of the total 430 residents in the institute, 255 (59%) responded to the survey. Specifically, participation rate among SRs was 74% (121/164) compared with 50% among NSRs (134/266). Full details of participation and distribution rates per individual residency programs are provided in Tables S1-S2.

The mean age of the 255 participants was 27.3 years. And 123 of them (48.2%) were males, 90 (35.3%) were first year residents, 134 (52.5%) were non-surgical residents and 121 (47.5%) were surgical residents. Table 1 shows the participants’ full demographic data.

**Table 1:** Participants general demographic data

	Frequency	%
Gender		
Male	123	48.2
Female	132	51.8
Age		
24–27	158	62.0
28–31	75	29.4
Older than 31	22	8.6
Marital Status		
Single	167	65.5
Married	88	34.5
Year of residency		
First year	90	35.3
Second year	58	22.7
Third year	52	20.4
Fourth/Fifth year	55	21.6
Surgery vs Non-surgery		
Non-surgical residents	134	52.5
Surgical residents	121	47.5

### The effect of the pandemic on the clinical settings and teaching programs

#### The effect on the clinical settings and main teaching program

When comparing changes in the clinical settings between surgical and non-surgical programs, the proportion of NSRs reporting lock-down of the clinic sometimes was significantly higher than the SRs group (42.5% vs 26.4% respectively; V = .169 “small effect” (95% CI; .042–.292), *p* = .007). Similarly, the proportion reporting a decrease in the staff working in the clinic (52.2% vs 29.8%; V = .228 “small effect” (95% CI; .099–.345), *p* < .001), the proportion reporting limited personal protective equipment (PPE) (71.6% vs 56.2%; V = .161 “small effect” (95% CI; .035–.281), *p* = .01), and the proportion reporting a delay in patients’ visitation to the clinic or emergency department due to fear of getting infected (57.5% vs 39.7%; V = .178 “small effect” (95% CI; .042–.297), *p* = .005) was higher among non-surgical residents. A significant difference was also detected in the effect on the number of on-call duties per month (V = .289 “small effect” (95% CI; .184–.403), *p* < .0001), with the most common answer in both groups being a “no change” (50.7% in the NSRs vs 64.5% in the SRs), while the second most common answer was an “increase” for the NSRs group (35.8% vs 11.5%), and a “decrease” for the SRs group (24% vs 13.5%).

As for the effect on the teaching programs, the proportion of NSRs reporting a shift toward online learning was significantly higher than that of the SRs group (53% vs 30.1%; V = .226 “small effect” (95% CI; .114–.350), *p* < .001).

And finally, the proportion of NSRs reporting participating in nasopharyngeal swab sampling for patients was also significantly higher than that of the SRs group (67.1% vs 34.7%; V = .324 “medium effect” (95% CI; .203–.442), *p* < .0001).

All questions, comparisons, and effect sizes related to the clinical settings and teaching programs are available in Table S3. Main results are presented in Table 2*.*

**Table 2:** Effect of the pandemic on the clinical settings and teaching programs

			Non-surgical residents (%)	Surgical residents (%)	
		Total (%)	134	121	*P*-value
Year of residency	First year	90 (35)	50 (37)	40 (33)	.108
	Second year	58 (23)	32 (24)	26 (22)	
	Third year	52 (20)	31 (23)	21 (17)	
	Fourth/fifth year	55 (22)	21 (16)	34 (28)	
Effect of the pandemic on the clinic: (yes)^a^					
a. Lock-down of the clinic sometimes		89 (35)	57 (43)	32 (26)	**.007**
b. Decrease in the number of patients per day		96 (38)	45 (34)	51 (42)	.159
c. Decrease in the number of staff working at the clinic		106 (42)	70 (52)	36 (30)	**<.001**
d. Limited personal protective equipment		164 (64)	96 (72)	68 (56)	**.010**
e. None of the above		23 (9)	12 (9)	11 (9)	.970
1) Effect on number of on-calls per month	Decreased.	47 (19)	18 (13)	29 (24)	**<.0001**
	Has not changed	146 (57)	68 (51)	78 (65)	
	Increased	62 (24)	48 (36)	14 (12)	
2) Noticing a delay in patients’ visitations to clinics or emergency departments due to patients’ fear from getting infected by COVID-19	Yes	125 (49)	77 (58)	48 (40)	**.005**
3) Noticing a decrease in the number of admissions during the pandemic	Yes	114 (45)	56 (42)	58 (48)	.325
Effect of the pandemic on the teaching program: (yes)^a^					
a. Less numbers of rounds		94 (37)	47 (35)	47 (39)	.533
b. Less numbers of lectures & seminars		183 (72)	100 (75)	83 (69)	.285
c. Shifting to online learning/meeting		108 (42)	71 (53)	37 (31)	**<.001**
d. Less numbers of grand rounds/lectures		115 (45)	68 (51)	47 (39)	.056
e. None of the above		12 (5)	8 (6)	4 (3)	.316
4) COVID-19 positive cases in the department/ward	Yes	211 (83)	105 (78)	106 (88)	.051
5) Participating in Nasopharyngeal swab sampling for patients	Yes	132 (52)	90 (67)	42 (35)	**<.0001**

^a^ Multiple selection questions

#### The effect on the surgical training program

**Fig. 1:**
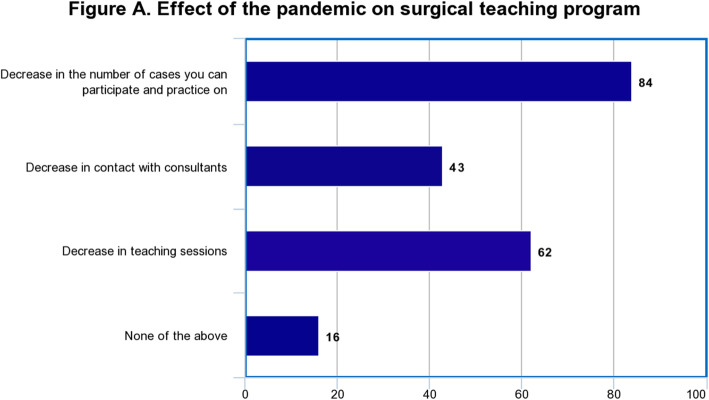
Effect of the pandemic on surgical teaching program

Regarding the surgical training program, when SRs were asked about a reduction in the number of elective surgeries, 63 (52.1%) provided a positive answer. In addition, in a multiple-selection question, 84 (69.4%) reported a decreased in the number of surgical cases they can participate and practice on, 43 (35.5%) reported a decrease in contact with the consultants, 62 (51.2%) reported a decrease in teaching sessions, and finally 16 (13.2%) had an answer of “none of the above”. Results of this question are represented in Fig. 1.

### The effect of the pandemic on residents’ mental health

First, a significant difference in gender between the two groups was detected, with the NSRs group having a higher proportion of females (60% vs 43.8%; *p* = .016).

When comparing the effect of the pandemic on the residents themselves between both groups, the proportion of residents reporting getting infected by COVID- 19 was significantly higher in the SRs group (9.9% vs 3.7%; V = .124 “small effect” (95% CI; .017–.229), *p* = .048). In addition, 113 (84%) residents in the NSRs group, and 98 (81%) residents in the SRs group reported a direct contact with COVID-19 cases. A statistically significant difference was found when they were asked about their relation to those infected people (patients, a co-worker, or both) (V = .193 “small effect” (95% CI; .089–.343), *p* = .020), with the most common answer in both groups being “both” (66.4% of the NSRs vs 49% of the SRs), while the second most common answer was “patients” in the NSRs group (20.4% vs 24.5%), and “co-workers” in the SRs group (26.5% vs 13.2%).

When asked about being updated about this pandemic protocol, a significant difference was also found (V = .210 “small effect” (95% CI; .105–.333), *p* = .004), with the most common answer in the NSRs group being “yes” (59% vs 38%) and the most common answer in the SRs group being “Not always” (44.6% vs 30.6%), while the remaining in both groups accounts for the “No” answer (10.4% of the NSRs vs 17.4% of the SRs). In addition, the proportion of residents who reported being trained to protect themselves against the virus spread was significantly higher in the NSRs group (56.7% vs 37.2%; V = .195 “small effect” (95% CI; .074–.307), *p* = .002). Similarly, the proportion of residents who reported being trained to protect others against the virus spread (54.5% vs 40.5%; V = .140 “small effect” (95% CI; .025–.256), *p* = .026) was also significantly higher in the NSRs group.

Regarding the effect on the residents’ mental health, when testing the internal consistency of the Likert-type questions addressing mental health, a Cronbach’s alpha of .802 (Good) was found.

When asked about feeling anxious about the pandemic in general based on a 4-point Likert scale, the median was 2 (2–3 IQR) in the NSRs group, vs 2 (1–2 IQR) in the SRs group, and the difference in distributions between the two groups was statistically significant, with the NSRs being more likely to feel anxious (r = 0.13 “small effect” (95% CI; 0.007–0.249), *p* = .044). Similarly, the proportion of residents who reported feeling anxious about an inadequacy of protective equipment in the work area was significantly greater in the NSRs group (90.3% vs 75.2%; V = .201 “small effect” (95% CI; .078–.313), *p* = .001), as well as the proportion of residents who reported feeling increased stress and anxiety between colleagues being also significantly higher in the NSRs group (88.1% vs 76%; V = .158 “small effect” (95% CI; .032–.279), *p* = .012).

As for the fear of getting infected, when asked on the 4-point Likert scale, the median was 2 (1–3 IQR) in the NSRs group, vs 2 (1–2 IQR) in the SRs group, and the difference in distributions between the two groups was statistically significant, with the NSRs being more likely to be afraid as well (*r* = 0.123 “small effect” (95% CI; 0–0.242), *p* = .049).

Effect sizes of all comparisons are available in Table S4. Main results are presented in Table 3.

**Table 3:** Effect of the pandemic on residents’ mental health

			Non-surgical residents (%)	Surgical residents (%)		
		Total (%)	134	121	*P*-value	
Gender	Male	123 (48)	55 (41)	68 (56)	**.016**	
	Female	132 (52)	79 (59)	53 (44)		
6) Isolation due to contact with COVID-19 positive cases	No	193 (76)	107 (80)	86 (71)	.186	
	Yes, I took less than a week off	40 (16)	19 (14)	21 (17)		
	Yes, I took a week or more off	22 (8)	8 (6)	14 (12)		
7) Number of getting personal screening or a diagnostic swab for COVID-19	Zero	75 (29)	38 (28)	37 (31)	.916	
	Once	91 (36)	48 (36)	43 (35)		
	More than once	89 (35)	48 (36)	41 (34)		
8) Getting infected by COVID-19	Yes	17 (7)	5 (4)	12 (10)	**.048**	
9) If yes, were you diagnosed by showing first:	Symptoms	10 (59)	3 (60)	7 (58)	1.00	
	Positive swab	7 (41)	2 (40)	5 (42)		
10) Having a direct contact with COVID-19 positive cases	Yes	211 (83)	113 (84)	98 (81)	.481	
11) If yes, were they co-workers or patients?	Patients	47 (22)	23 (21)	24 (25)	**.020**	
	Co-workers	41 (20)	15 (13)	26 (26)		
	Both	123 (58)	75 (66)	48 (49)		
12) Being updated about this pandemic protocol	Yes	125 (49)	79 (59)	46 (38)	**.004**	
	Not always	95 (37)	41 (31)	54 (45)		
	No	35 (14)	14 (10)	21 (17)		
13) Changing the work area inside or outside the hospital due to Corona Pandemic Protocol	Yes	85 (33)	44 (33)	41 (34)	.859	
14) Being trained on how to protect self against COVID-19 spread	Yes	121 (48)	76 (57)	45 (37)	**.002**	
15) Being trained on how to protect others against COVID-19 spread	Yes	122 (48)	73 (55)	49 (41)	**.026**	
16) Being anxious about the pandemic	Median (IQR)	2 (2–2)	2 (2–3)	2 (1–2)	.044**	
	Always	61 (24)	39 (29)	22 (18)		
	Most of the time	133 (52)	67 (50)	66 (54)		
	Rarely	56 (22)	26 (19)	30 (25)		
	Never	5 (2)	2 (2)	3 (3)		
17) Having adequate personal protective equipment in the work area for Corona Pandemic	Yes	65 (26)	27 (20)	38 (31)	.117	
	Not always	98 (38)	56 (42)	42 (35)		
	No	92 (36)	51 (38)	41 (34)		
18) Being anxious about an inadequacy of protective equipment in the work area	Yes	212 (83)	121 (90)	91 (75)	**.001**	
19) Increased stress and anxiety between colleagues	Yes	210 (82)	118 (88)	92 (76)	**.012**	
20) Complaining from Depression symptoms due to the Pandemic	Median	1 (1–2)	2 (1–2)	1 (1–2)	.055**	
	Always	36 (14)	17 (13)	19 (16)		
	Most of the time	89 (35)	61 (45)	28 (23)		
	Rarely	82 (32)	33 (25)	49 (40)		
	Never	48 (19)	23 (17)	25 (21)		
21) Fear of getting infected	Median (IQR)	2 (1–3)	2 (1–3)	2 (1–2)	.049**	
	Always	79 (31)	52 (39)	27 (22)		
	Most of the time	102 (40)	45 (33)	57 (47)		
	Rarely	59 (23)	29 (22)	30 (25)		
	Never	15 (6)	8 (6)	7 (6)		
22) Fear of death due to the pandemic	Median (IQR)	1 (1–2)	1 (1–2)	1 (1–2)	.341**	
	Always	43 (17)	26 (19)	17 (14)		
	Most of the time	71 (28)	39 (29)	32 (26)		
	Rarely	106 (41)	49 (37)	57 (47)		
	Never	35 (14)	20 (15)	15 (13)		
23) Number of family members living in the same house	Zero	29 (11)	11 (8)	18 (15)	.104	
	One	22 (9)	10 (8)	12 (10)		
	Two	25 (10)	18 (13)	7 (6)		
	Three	35 (14)	16 (12)	19 (15)		
	Four or more	144 (56)	79 (59)	65 (54)		
24) If present, does family members in the previous question include seniors (older than 60 years old members)	Yes	120 (53)	67 (55)	53 (52)	.651	
25) One of the family members getting infected	Yes	28 (12)	20 (16)	8 (8)	.054	
26) Feeling guilt or fear of spreading COVID-19 from your work area to family members?	Median (IQR)	3 (2–3)	3 (3–3)	3 (2–3)	.077**	
	Always	180 (70)	101 (75)	79 (65)		
	Most of the time	46 (18)	20 (15)	26 (22)		
	Rarely	12 (5)	7 (5)	5 (4)		
	Never	17 (7)	6 (5)	11 (9)		
27) Feeling safe during the pandemic	Median (IQR)	1 (0–1)	1 (0–1)	1 (0–1)	.546**	
	Always	6 (2)	2 (2)	4 (3)		
	Most of the time	23 (9)	9 (7)	14 (12)		
	Rarely	114 (45)	64 (47)	50 (41)		
	Never	112 (44)	59 (44)	53 (44)		
28) Feeling that family members are safe during the pandemic	Median (IQR)	1 (0–1)	1 (0–1)	1 (0–1)	.108**	
	Always	2 (1)	1 (1)	1 (1)		
	Most of the time	25 (11)	10 (8)	15 (14)		
	Rarely	96 (43)	51 (41)	45 (44)		
	Never	103 (46)	61 (50)	42 (41)		
29) Staying away from the family in order to protect them	Yes	189 (74)	105 (78)	84 (69)	.104	
30) Changing or considering changing your specialty in order to protect yourself or your family from COVID-19	Yes	38 (15)	19 (14)	19 (16)	.733	
31) Decreased available time for other activities outside the hospital	Median (IQR)	2 (2–3)	2 (2–3)	2 (2–3)	.504**	
	Always	115 (45)	60 (46)	55 (45)		
	Most of the time	110 (43)	64 (38)	46 (43)		
	Rarely	23 (9)	8 (12)	15 (9)		
	Never	7 (3)	2 (4)	5 (3)		
32) Missing an event/activity due to COVID-19 Pandemic	Yes	243 (95)	128 (96)	115 (95)	.856	
33) Facing any limitations in coming to work because of the Lock-down	Yes	142 (56)	77 (58)	65 (54)	.548	
34) Other work-related effects of the lock-down: (yes)*						
a. Absences from work		35 (14)	19 (14)	16 (13)	.825	
b. Delay in arriving to the work		86 (34)	47 (35)	39 (32)	.632	
c. Delay in leaving your work		98 (38)	53 (40)	45 (37)	.699	
d. More responsibilities at work		163 (64)	91 (68)	72 (60)	.163	
e. None of the above		41 (16)	19 (14)	22 (18)	.385	

* Multiple selection questions

** *p*-values were computed using the Mann Whitney U test

## Discussion

This study presents a comprehensive insight on the burden of the ongoing COVID-19 pandemic on residents’ health and the workflow of their training programs at a major tertiary hospital in North Jordan.

The results of this survey study indicate the crucial restraints the pandemic had on residency programs at KAUH. In this regard, the closure of outpatient clinics as well as the delay of most elective and non-urgent surgical and medical procedures had significantly reduced residents’ clinical interaction as this was the universal approach in healthcare prioritization, Farid et al. reported a major reduction in global surgical activity in a surgery department at Belgium [17]. These workspace restraints were coupled with the mental and physical burden the pandemic has left on residents and as a result in a way or another this has brought light to the adopted fragile support systems for healthcare workers in general. In this context, we definitely cannot ignore the uniqueness of the circumstances and how this opens a door for experimentation before identifying the optimum adaptation methods, yet there are established mainstays in dealing with such tolls.

On the other side, positive outcomes of the current crises are also worth mentioning and they include the implementation of effective distant learning methods such as online seminars, recorded or live-streamed didactic lectures as well as the introduction of telemedicine in a comforting and efficient method to the patients and healthcare providers. Overall, most of these online-based methods will transcend beyond the pandemic as they have proven to be effective time-efficient tools in education and in some instances in health-related services as well [18–20].

In order to further understand the effect of the current exceptional circumstances, our sample was stratified according to the nature of the residency program, as residents in surgical programs undergo a more hands-on approach in the day-to-day practice and it is established that COVID-19 is more likely to spread in close human interactions [21]. When considering the effect of the pandemic on the clinical settings, “limited Personal Protective Equipment” was the most frequent complaint in our cohort followed by “the decrease in the number of staff working at the clinic”. In both instances, NSRs more frequently reported these complaints. This could be explained by the increased COVID-19 related workload on NSRs as they interact more with COVID-19 patients upon ICU or inpatient admission. This is further highlighted by the significantly increased number of on call duties per month as well as the participation in nasopharyngeal swab sampling for patients in the NSRs cohort relative to SRs. A realistic solution to limit the ramifications of increased workload would be to incorporate medical students into the healthcare workforce as suggested by Agarwal et al. [7]. Yet, such crucial move would require immense sufficient training beforehand [22].

In our sample almost half of the residents were trained on how to protect themselves and others against COVID-19 spread, this is similar to what was reported in a similar Saudi study [9]. This study also reported a lower COVID-19 infection rate compared to our sample. Yet, it is worth noting that the time span of both studies is different; Balhareth et al. collected responses in the period between April 23rd and May 6th 2020 and Saudi Arabia had a total of 31,938 cases by May 6th 2020 whereas responses’ collection in our study was done between October 30th and November 8th 2020 and Jordan had a total of 109,321 cases by November 8th as there was a surge in cases in that period [5, 9, 23].

Interestingly in our cohort, SRs were infected by COVID-19 more frequently than NSRs which could appear to be counter intuitive for a moment since NSRs are more frequently to interact with COVID-19 patients. However, we believe the psychological aspect of cautious practice in this scenario plays a crucial role, since SRs could underestimate the importance of key protective measures due to out-of-place ease when interacting with surgical patients when in fact those patients need more caution since they can be asymptomatic or silent COVID-19 patients. This difference could also be explained by the higher percentage of NSRs being both “updated with the pandemic protocol” and “trained on how to protect themselves against COVID-19 spread”. This again highlights a potential worrying cascade of events that starts with the frontline involvement of COVID-19 care being non-surgical healthcare personnel and as a result surgery-related healthcare worker will be less updated and perhaps less aware of the essential protective measures.

The more active role of NSRs in our cohort is in return reflected on the mental status of residents, as NSRs have answered the question “Being anxious about the pandemic” with “Always” more frequently than SRs. In this regard, the question “being anxious about an inadequacy of protective equipment in work and the question “Increased stress and anxiety between colleagues” confirm this conclusion, since a significant difference was observed in the percentage of (Yes) answers in both questions. Aljehani et al. found that almost half of screened general surgery residents during the current pandemic had a positive score on the screening tool for generalized anxiety disorder (GAD) [24]. Considering these alarming high percentages of stress and anxiety among residents specifically and healthcare workers generally, increased institutional efforts should be invested in approaching this burden to avoid further exacerbation of this issue. Suggested measures to manage this problem include providing counseling sessions, anonymous crisis hotlines and holding seminars on effective stress coping mechanisms [25]. It is also critical not to underestimate the culture that we foster within our medical institutes as they can be a catalyst in either building or breaking trust and empathy between the healthcare workforce [26].

The current study presents relevant meaningful data that describes and reflects the status of a population that is not extensively studied and even in some instances neglected or given marginal attention. In addition, this study takes into consideration the nature of residency programs (surgical vs medical) when evaluating the repercussions of the pandemic on residents, this in return yields more appropriate descriptive results. Findings from this study will present a primordial core upon which future informed decisions can be made, in this very instance the value of such insight is further appreciated since devastating pandemics like the current are quite infrequent and simply unprecedented. Hence, capitalizing on learning as much as possible from such conditions is a necessity that is indeed time restricted.

The main limitation in our study is the limited generalizability of the results as the study was implemented at a single tertiary hospital in North of Jordan that was allocated to be one of a few COVID-19 testing and admission centers in the country. In addition, being an online survey questionnaire might have contributed to response bias that could not be managed and controlled adequately. Additionally, some highly significant *P* values should not be misinterpreted for exaggerated effects as most of the effect sizes were relatively small.

National multi-center studies involving larger samples that cover primary, secondary, and tertiary healthcare centers are still needed to confirm these findings on a large scale. In the context of a healthcare-related crisis we recommend conducting a comprehensive preparation program at the national level that covers crisis management as well as self-care protocol for healthcare workers, an interventional study assessing the pre- and post-outcomes off such program would provide a realistic in depth look into the dimensions of such solution.

## Conclusion

The burden of this crisis caused by COVID-19 pandemic on the mental health status of residents is very alarming and definitely should not left for disorganized efforts. As this requires a structured system that provides residents with psychological counseling and support needs. Without any doubt, the pandemic has some critical implications on the flow of residency training programs. This in return calls out for collaborative efforts from healthcare institutes to adapt and adopt smart educational techniques to compensate for such limitations. In addition, training and updating residents and healthcare workers should be given a priority in any healthcare-related crisis as they are the first line fighters in such exceptional circumstances.

## Supplementary Information

**Additional file 1: Tables S1.** Participation rates per individual residency programs. **Tables S2.** Distribution of participants upon residency programs. **Table S3.** All questions and results related to the effect on clinical settings and teaching programs. **Table S4.** All results related to the effect on the residents’ mental health.

## Data Availability

The datasets used and/or analysed during the current study are available from the corresponding author on reasonable request.
