# Supplementary material for: The impact of COVID-19 pandemic on training and mental health of residents: a cross-sectional study
Source: BMC Med Educ. 2021 Apr 13;21:208. doi: 10.1186/s12909-021-02655-2 (PMC8041616; doi:10.1186/s12909-021-02655-2)
Supplement: Supplementary file 1 — Additional file 1: Tables S1. Participation rates per individual residency programs. Tables S2. Distribution of participants upon residency programs. Table S3. All questions and results related to the effect on clinical settings and teaching programs. Table S4. All results related to the effect on the residents’ mental health. [file 12909_2021_2655_MOESM1_ESM.docx]

**The impact of COVID-19 pandemic on training and mental health of residents: a cross-sectional study**

**Eman Alshdaifat:** Department of Obstetrics and Gynecology, Faculty of medicine, Yarmouk University, Irbid- Jordan. Email: [eman.shdaifat@yu.edu.jo](mailto:eman.shdaifat@yu.edu.jo).

**Amer Sindiani:** Department of Obstetrics and Gynecology, Faculty of medicine, Jordan University of Science and Technology, Irbid- Jordan. Email: [amsindiani0@just.edu.jo](mailto:amsindiani0@just.edu.jo).

**Wasim Khasawneh:** Department of Pediatrics and Neonatology Faculty of medicine, Jordan University of Science and Technology, Irbid- Jordan. Email: [wakhasawneh@just.edu.jo](mailto:wakhasawneh@just.edu.jo).

**Omar Abu-Azzam:** Department of Obstetrics and Gynecology, Faculty of medicine, Mutah University, Al-karak- Jordan. Email: [oabuazzam@yahoo.com](mailto:oabuazzam@yahoo.com).

**Aref Qarqash:** Medical students, Faculty of medicine, Jordan University of Science and Technology, Irbid- Jordan. Email: [aaqarqash1819@med.just.edu.jo](mailto:aaqarqash1819@med.just.edu.jo).

**Hassan Abushukair:** Medical students, Faculty of medicine, Jordan University of Science and Technology, Irbid- Jordan. Email: [hmabushukair182@med.just.edu.jo](mailto:hmabushukair182@med.just.edu.jo).

**Nail Obeidat:** Department of Obstetrics and Gynecology, Faculty of medicine, Jordan University of Science and Technology, Irbid- Jordan. Email: [naobeidat@just.edu.jo](mailto:naobeidat@just.edu.jo).

***Corresponding author**: Eman Alshdaifat, Department of Obstetrics and Gynecology, Faculty of medicine, Yarmouk University P.O.Box: (566) Irbid 21163- Jordan, Tel.: +962795780335

Email:eman.shdaifat@yu.edu.jo

Tables S1: Participation rates per individual residency programs

|  | | Total | Responded (%) |
| --- | --- | --- | --- |
| Over-all |  | 430 | 255 (59) |
| General specialty | Surgical | 164 | 121(74) |
|  | Non-surgical | 266 | 134 (50) |
| Detailed  Specialty | Anesthesia | 34 | 12(35) |
|  | Dermatology | 13 | 9 (69) |
|  | Emergency medicine | 18 | 2 (11) |
|  | ENT surgery | 11 | 11 (100) |
|  | Family medicine | 40 | 32 (80) |
|  | General Surgery | 59 | 44 (75) |
|  | Internal medicine | 59 | 37 (63) |
|  | Neuromedicine | 21 | 3 (14) |
|  | Neurosurgery | 9 | 5 (56) |
|  | Obstetrics & Gynecology | 39 | 38 (97) |
|  | Opthalmology | 20 | 9 (45) |
|  | Orthopedic | 14 | 9 (64) |
|  | Pathology | 13 | 3 (23) |
|  | Pediatric | 24 | 21 (88) |
|  | Psychiatry | 10 | 4 (40) |
|  | Radiology | 34 | 11 (32) |
|  | Urology | 12 | 5 (42) |

Tables S2: Distribution of participants upon residency programs

|  | | Frequency | % |
| --- | --- | --- | --- |
| Total |  | 255 | 100 |
| Specialty | Anesthesia | 12 | 4.8 |
|  | Dermatology | 9 | 3.5 |
|  | Emergency Doctor | 2 | 0.8 |
|  | Ent resident | 11 | 4.3 |
|  | Family medicine resident | 32 | 12.5 |
|  | General Surgery resident | 44 | 17.3 |
|  | Internal medicine resident | 37 | 14.5 |
|  | Neuromedicine resident | 3 | 1.2 |
|  | Neurosurgery resident | 5 | 2.0 |
|  | Obstetrics & Gynecology resident | 38 | 14.9 |
|  | Ophthalmology resident | 9 | 3.5 |
|  | Orthopedic resident | 9 | 3.5 |
|  | Pathology resident | 3 | 1.2 |
|  | Pediatric resident | 21 | 8.2 |
|  | Psychiatry resident | 4 | 1.6 |
|  | Radiology resident | 11 | 4.3 |
|  | Urology resident | 5 | 2.0 |
| Surgery vs Non-surgery | Non-surgical residents | 134 | 52.5 |
|  | Surgical residents | 121 | 47.5 |

Table S3: All questions and results related to the effect on clinical settings and teaching programs

|  | | |  | Non-surgical residents (%) | Surgical residents (%) |  |  | |  |
| --- | --- | --- | --- | --- | --- | --- | --- | --- | --- |
|  |  | Total (%) | | 134 | 121 | P-value | | Effect size (V) (95% CI) | |
| Year of residency | First year | 90 (35) | | 50 (37) | 40 (33) | .108 | |  | |
|  | Second year | 58 (23) | | 32 (24) | 26 (22) |  | |  | |
|  | Third year | 52 (20) | | 31 (23) | 21 (17) |  | |  | |
|  | Fourth/fifth year | 55 (22) | | 21 (16) | 34 (28) |  | |  | |
| 1. Effect of the pandemic on the clinic: (yes) | |  | |  |  |  | |  | |
| Lock-down of the clinic sometimes |  | 89 (35) | | 57 (43) | 32 (26) | **.007** | | .169 (042-.292) | |
| Decrease in the number of patients per day |  | 96 (38) | | 45 (34) | 51 (42) | .159 | | .088 (.006-.218) | |
| Decrease in the number of staff working at the clinic |  | 106 (42) | | 70 (52) | 36 (30) | **<.001** | | .228 (.099-.345) | |
| Limited personal protective equipment |  | 164 (64) | | 96 (72) | 68 (56) | **.010** | | .161 (.035-.281) | |
| None of the above |  | 23 (9) | | 12 (9) | 11 (9) | .970 | | .002 (.002-.133) | |
| 1. Effect on number of on calls per month | Decreased. | 47 (19) | | 18 (13) | 29 (24) | **<.0001** | | .289 (.184-.403) | |
|  | Has not changed | 146 (57) | | 68 (51) | 78 (65) |  | |  | |
|  | Increased | 62 (24) | | 48 (36) | 14 (12) |  | |  | |
| 1. Noticing a delay in patients' visitations to clinics or emergency departments due to patients' fear from getting infected by COVID-19 | Yes | 125 (49) | | 77 (58) | 48 (40) | **.005** | | .178 (.042-.297) | |
| 1. Do you think non-COVID patients were negatively affected during the pandemic? | Yes | 227 (89) | | 124 (93) | 103 (85) | .059 | | .118 (.011-.227) | |
| 1. Noticing a decrease in the number of admissions during the pandemic | Yes | 114 (45) | | 56 (42) | 58 (48) | .325 | | .062 (.003-.182) | |
| 1. The number of staff working in your department during the pandemic has: | Decreased | 121 (48) | | 68 (51) | 53 (44) | .311 | | .097 (.025-.229) | |
|  | Not changed | 125 (49) | | 60 (45) | 65 (54) |  | |  | |
|  | Increased | 9 (3) | | 6 (5) | 3 (3) |  | |  | |
| 1. Effect of the pandemic on the teaching program: (yes) | |  | |  |  |  | |  | |
| Less numbers of rounds |  | 94 (37) | | 47 (35) | 47 (39) | .533 | | .039 (.003-.163) | |
| Less numbers of lectures & seminars |  | 183 (72) | | 100 (75) | 83 (69) | .285 | | .067 (.003-.179) | |
| Shifting to online learning/meeting |  | 108 (42) | | 71 (53) | 37 (31) | **<.001** | | .226 (.114-.350) | |
| Less numbers of grand rounds/lectures |  | 115 (45) | | 68 (51) | 47 (39) | .056 | | .119 (.012-.237) | |
| None of the above |  | 12 (5) | | 8 (6) | 4 (3) | .316 | | .063 (.003-.181) | |
| 1. COVID-19 positive cases in the department/ward | Yes | 211 (83) | | 105 (78) | 106 (88) | .051 | | .122 (.013-.229) | |
| 1. Participating in Nasopharyngeal swab sampling for patients | Yes | 132 (52) | | 90 (67) | 42 (35) | **<.0001** | | .324 (.203-.442) | |

Table S4: All results related to the effect on the residents’ mental health

|  | | | |  | | Non-surgical residents (%) | Surgical residents (%) |  |  |  |
| --- | --- | --- | --- | --- | --- | --- | --- | --- | --- | --- |
|  |  | | Total (%) | | 134 | | 121 | P-value | Effect size (V, r) (95% CI) |  |
| Gender | Male | | 123 (48) | | 55 (41) | | 68 (56) | **.016** |  |  |
|  | Female | | 132 (52) | | 79 (59) | | 1. (44) |  |  |  |
| 1. Isolation due to contact with COVID-19 positive cases | No | | 193 (76) | | 107 (80) | | 86 (71) | .186 | .115 (.035-.238) |  |
|  | Yes, I took less than a week off | | 40 (16) | | 19 (14) | | 21 (17) |  |  |  |
|  | Yes, I took a week or more off | | 22 (8) | | 8 (6) | | 14 (12) |  |  |  |
| 1. Number of getting personal screening or a diagnostic swab for COVID-19 | Zero | | 75 (29) | | 38 (28) | | 37 (31) | .916 | .026 (.014-.177) |  |
|  | Once | | 91 (36) | | 48 (36) | | 43 (35) |  |  |  |
|  | More than once | | 89 (35) | | 48 (36) | | 41 (34) |  |  |  |
| 1. Getting infected by COVID-19 | Yes | | 17 (7) | | 5 (4) | | 12 (10) | **.048** | .124 (.017-.229) |  |
| 1. If yes, were you diagnosed by showing first: | Symptoms | | 10 (59) | | 3 (60) | | 7 (58) | 1.00 | .015 (.015-.528) |  |
|  | Positive swab | | 7 (41) | | 2 (40) | | 5 (42) |  |  |  |
| 1. Having a direct contact with COVID-19 positive cases | Yes | | 211 (83) | | 113 (84) | | 98 (81) | .481 | .044 (.002-.163) |  |
| 1. If yes, were they co-workers or patients? | Patients | | 47 (22) | | 23 (21) | | 24 (25) | **.020** | .193 (.089-.343) |  |
|  | Co-workers | | 41 (20) | | 15 (13) | | 26 (26) |  |  |  |
|  | Both | | 123 (58) | | 75 (66) | | 48 (49) |  |  |  |
| 1. Being updated about this pandemic protocol | Yes | | 125 (49) | | 79 (59) | | 46 (38) | **.004** | .210 (.105-.333) |  |
|  | Not always | | 95 (37) | | 41 (31) | | 54 (45) |  |  |  |
|  | No | | 35 (14) | | 14 (10) | | 21 (17) |  |  |  |
| 1. Changing the work area inside or outside the hospital due to Corona Pandemic Protocol | Yes | | 85 (33) | | 44 (33) | | 41 (34) | .859 | .011 (.001-.150) |  |
| 1. Being trained on how to protect self against COVID-19 spread | Yes | | 121 (48) | | 76 (57) | | 45 (37) | **.002** | .195 (.074-.307) |  |
| 1. Being trained on how to protect others against COVID-19 spread | Yes | | 122 (48) | | 73 (55) | | 49 (41) | **.026** | .140 (.025-.256) |  |
| 1. Being anxious about the pandemic | Median (IQR) | | 2 (2-2) | | 2 (2-3) | | 2 (1-2) | **.044*** | 0.13 (0.007-0.249)** |  |
|  | Always | | 61 (24) | | 39 (29) | | 22 (18) |  |  |  |
|  | Most of the time | | 133 (52) | | 67 (50) | | 66 (54) |  |  |  |
|  | Rarely | | 56 (22) | | 26 (19) | | 30 (25) |  |  |  |
|  | Never | | 5 (2) | | 2 (2) | | 3 (3) |  |  |  |
| 1. Having adequate personal protective equipment in the work area for Corona Pandemic | Yes | | 65 (26) | | 27 (20) | | 38 (31) | .117 | .130 (.042-.275) |  |
|  | Not always | | 98 (38) | | 56 (42) | | 42 (35) |  |  |  |
|  | No | | 92 (36) | | 51 (38) | | 41 (34) |  |  |  |
| 1. Being anxious about an inadequacy of protective equipment in the work area | Yes | | 212 (83) | | 121 (90) | | 91 (75) | **.001** | .201 (.078-.313) |  |
| 1. Increased stress and anxiety between colleagues | Yes | | 210 (82) | | 118 (88) | | 92 (76) | **.012** | .158 (.032-.279) |  |
| 1. Complaining from Depression symptoms due to the Pandemic | Median | | 1 (1-2) | | 2 (1-2) | | 1 (1-2) | .055* | 0.12 (0-0.24)** |  |
|  | Always | | 36 (14) | | 17 (13) | | 19 (16) |  |  |  |
|  | Most of the time | | 89 (35) | | 61 (45) | | 28 (23) |  |  |  |
|  | Rarely | | 82 (32) | | 33 (25) | | 49 (40) |  |  |  |
|  | Never | | 48 (19) | | 23 (17) | | 25 (21) |  |  |  |
| 1. Fear of getting infected | Median (IQR) | | 2 (1-3) | | 2 (1-3) | | 2 (1-2) | **.049*** | 0.123 (0-0.242)** |  |
|  | Always | | 79 (31) | | 52 (39) | | 27 (22) |  |  |  |
|  | Most of the time | | 102 (40) | | 45 (33) | | 57 (47) |  |  |  |
|  | Rarely | | 59 (23) | | 29 (22) | | 30 (25) |  |  |  |
|  | Never | | 15 (6) | | 8 (6) | | 7 (6) |  |  |  |
| 1. Fear of death due to the pandemic | Median (IQR) | | 1 (1-2) | | 1 (1-2) | | 1 (1-2) | .341* | 0.06 (-0.06-0.18)** |  |
|  | Always | | 43 (17) | | 26 (19) | | 17 (14) |  |  |  |
|  | Most of the time | | 71 (28) | | 39 (29) | | 32 (26) |  |  |  |
|  | Rarely | | 106 (41) | | 49 (37) | | 57 (47) |  |  |  |
|  | Never | | 35 (14) | | 20 (15) | | 15 (13) |  |  |  |
| 1. Number of family members living in the same house | Zero | | 29 (11) | | 11 (8) | | 18 (15) | .104 | .174 (.104-.321) |  |
|  | One | | 22 (9) | | 10 (8) | | 12 (10) |  |  |  |
|  | Two | | 25 (10) | | 18 (13) | | 7 (6) |  |  |  |
|  | Three | | 35 (14) | | 16 (12) | | 19 (15) |  |  |  |
|  | Four or more | | 144 (56) | | 79 (59) | | 65 (54) |  |  |  |
| 1. If present, does family members in the previous question include seniors (older than 60 years old members) | Yes | | 120 (53) | | 67 (55) | | 53 (52) | .651 | .030 (.002-.158) |  |
| 1. One of the family members getting infected | Yes | | 28 (12) | | 20 (16) | | 8 (8) | .054 | .128 (.015-.243) |  |
| 1. Feeling guilt or fear of spreading COVID-19 from your work area to family members? | Median (IQR) | | 3 (2-3) | | 3 (3-3) | | 3 (2-3) | .077* | 0.11 (-0.01-0.23)** |  |
|  | Always | | 180 (70) | | 101 (75) | | 79 (65) |  |  |  |
|  | Most of the time | | 46 (18) | | 20 (15) | | 26 (22) |  |  |  |
|  | Rarely | | 12 (5) | | 7 (5) | | 5 (4) |  |  |  |
|  | Never | | 17 (7) | | 6 (5) | | 11 (9) |  |  |  |
| 1. Feeling safe during the pandemic | Median (IQR) | | 1 (0-1) | | 1 (0-1) | | 1 (0-1) | .546* | 0.04 (-0.08-0.16)** |  |
|  | Always | | 6 (2) | | 2 (2) | | 4 (3) |  |  |  |
|  | Most of the time | | 23 (9) | | 9 (7) | | 14 (12) |  |  |  |
|  | Rarely | | 114 (45) | | 64 (47) | | 50 (41) |  |  |  |
|  | Never | | 112 (44) | | 59 (44) | | 53 (44) |  |  |  |
| 1. Feeling that family members are safe during the pandemic | Median (IQR) | | 1 (0-1) | | 1 (0-1) | | 1 (0-1) | .108* | 0.1 (-0.02-0.22)** |  |
|  | Always | | 2 (1) | | 1 (1) | | 1 (1) |  |  |  |
|  | Most of the time | | 25 (11) | | 10 (8) | | 15 (14) |  |  |  |
|  | Rarely | | 96 (43) | | 51 (41) | | 45 (44) |  |  |  |
|  | Never | | 103 (46) | | 61 (50) | | 42 (41) |  |  |  |
| 1. Staying away from the family in order to protect them | Yes | | 189 (74) | | 105 (78) | | 84 (69) | .104 | .102 (.009-.233) |  |
| 1. Changing or considering changing your specialty in order to protect yourself or your family from COVID-19 | Yes | | 38 (15) | | 19 (14) | | 19 (16) | .733 | .021 (.002-.150) |  |
| 1. Decreased available time for other activities outside the hospital | Median (IQR) | | 2 (2-3) | | 2 (2-3) | | 2 (2-3) | .504* | 0.04 (-0.08-0.16)** |  |
|  | Always | | 115 (45) | | 60 (46) | | 55 (45) |  |  |  |
|  | Most of the time | | 110 (43) | | 64 (38) | | 46 (43) |  |  |  |
|  | Rarely | | 23 (9) | | 8 (12) | | 15 (9) |  |  |  |
|  | Never | | 7 (3) | | 2 (4) | | 5 (3) |  |  |  |
| 1. Missing an event/activity due to COVID-19 Pandemic | Yes | | 243 (95) | | 128 (96) | | 115 (95) | .856 | .011 (.003-.141) |  |
| 1. Facing any limitations in coming to work because of the Lock-down | Yes | | 142 (56) | | 77 (58) | | 65 (54) | .548 | .038 (.003-.162) |  |
| 1. Other work-related effects of the lock-down (yes) | | |  | |  | |  |  |  |  |
| Absences from work | |  | 35 (14) | | 19 (14) | | 16 (13) | .825 | .014 (.002-.140) |  |
| Delay in arriving to the work | |  | 86 (34) | | 47 (35) | | 39 (32) | .632 | .030 (.002-.154) |  |
| Delay in leaving your work | |  | 98 (38) | | 53 (40) | | 45 (37) | .699 | .024 (.003-.159) |  |
| More responsibilities at work | |  | 163 (64) | | 91 (68) | | 72 (60) | .163 | .087 (.005-.213) |  |
| None of the above | |  | 41 (16) | | 19 (14) | | 22 (18) | .385 | .054 1(.002-.169) |  |

* p-values were computed using the Mann Whitney U test.

** Effect sizes are expressed as r statistics.
